# Cost Analysis of Multidose Drug Dispensing (MDD) System Implementation in a Community Pharmacy in Portugal

**DOI:** 10.3390/pharmacy13060175

**Published:** 2025-12-01

**Authors:** Ana Reis, Ângelo Jesus, Maria Luisa Martín

**Affiliations:** 1 LAQV/REQUIMTE, Escola Superior de Saúde, Instituto Politécnico do Porto, Rua Dr. António Bernardino de Almeida, 400, 4200-072 Porto, Portugal; 2 Grupo de Investigación en Fisiología y Farmacología, Facultad de Farmacia, Universidad de Salamanca, Campus Miguel de Unamuno s/n, 37007 Salamanca, Spain

**Keywords:** multidose drug dispensing, pharmacy services, economic evaluation, medication adherence, healthcare costs

## Abstract

Background: Community pharmacies are increasingly delivering structured services to support chronic disease management, such as Multidose Drug Dispensing (MDD). This strategy can improve adherence and safety, but evidence of its economic feasibility in Portuguese pharmacies remains limited. Objective: To estimate the cost of implementing and operating an MDD system in a community pharmacy, informing reimbursement models and policy. Methods: A micro-costing approach assessed fixed and variable expenses for serving polymedicated elderly patients. Costs were calculated in euros (2024/2025) and expressed per working day based on 253 annual preparation days. Results: First-year costs totaled €70,985.68, including €8184.00 for setup, €21,579.00 for supplies, and €41,222.68 for staff salaries. The daily operating cost was €280.58, with labour representing the major expense. A break-even analysis indicated sustainability with around 700 users at €10/month. Conclusion: Although requiring significant initial investment, MDD can become financially viable through scaling, workflow efficiency, and supportive reimbursement strategies.

## 1. Introduction

The Multidose Drug Dispensing (MDD) Service Model refers to a structured pharmaceutical service in which oral solid dosage forms are pre-packaged into individualized, time-specific doses according to a patient’s daily regimen. Core components include the use of automated or semi-automated packaging technologies, pharmacist validation, and periodic review of medication regimens. The target population typically comprises polymedicated older adults, especially those with chronic conditions or living in institutional settings [1,2,3,4,5,6,7,8,9,10].

Community pharmacies play an evolving role in delivering patient-centred services to address the challenges caused by chronic disease management and medication non-adherence. Multidose Drug Dispensing (MDD) systems offer a structured approach by pre-packaging oral solid medications into unit doses aligned with patients’ daily regimens. The potential of MDD to reduce medication errors, improve adherence, and support healthcare efficiency has been recognized in several European settings [1,2].

Non-adherence remains a major cause of suboptimal therapeutic outcomes and increased hospitalizations, particularly among older adults with multiple chronic conditions [3,4,5,6,7]. Pharmacy-led interventions, including medication reviews and MDD, have demonstrated effectiveness in mitigating these issues [8,9,10,11,12]. Portuguese community pharmacies, some of them already engaged in collabourative care models, are well-positioned to integrate MDD services, yet comprehensive cost evaluations are limited [13,14,15,16,17,18,19,20,21].

Despite the recognized potential of MDD, evidence regarding its economic and practical feasibility in real-world pharmacy settings remains scarce. Most existing studies have focused on clinical or adherence outcomes, often neglecting operational costs, workflow implications, and sustainability aspects [20,22,23,24,25,26,27,28,29,30,31]. Understanding the economic dimension is critical for informing pharmacy practice, policy decisions, and reimbursement frameworks, particularly in health systems under cost-containment pressures [32,33].

Furthermore, the growing burden of multimorbidity and polypharmacy in ageing populations makes the optimization of medication management increasingly urgent. In this context, MDD can contribute not only to safer dispensing processes but also to the redefinition of pharmacists’ professional roles from product suppliers to integrated members of multidisciplinary healthcare teams [10,19,21,34,35,36,37].

This study aims to estimate the full economic cost of MDD implementation in a Portuguese community pharmacy, offering evidence to inform healthcare stakeholders and guide potential reimbursement strategies.

## 2. Materials and Methods

In this study, the economic data were presented as illustrative of a single community pharmacy providing an operational MDD service for both institutionalized and community-dwelling elderly patients. Cost data were triangulated from multiple verified sources, including supplier catalogues, pharmacy accounting systems, and national collective wage agreements. Although a single pharmacy limits representativeness, cost categories and workflows are typical for Portuguese community settings, allowing cautious extrapolation. This analysis includes capital, operational, and human resource costs. However, indirect and long-term costs such as staff training, utilities, and maintenance are acknowledged as missing from the base model due to data unavailability. Their omission is expected to slightly underestimate total implementation costs. A detailed breakdown of cost categories—including capital expenditure, recurrent operational costs, and labour—was compiled from publicly available information on pharmacy-related websites, other healthcare providers, and an operational MDD service established in a pharmacy serving elderly and polymedicated patients, whether institutionalized or not. The websites and other sources consulted are based on publicly available data from several pharmacy distributors operating in Portugal, as well as general online retail platforms accessible worldwide.

Costs were calculated in euros for the year 2024/2025 and expressed per working day, assuming 253 days of annual MDD preparation, according to the Portuguese working-day calendar in the year 2025 (www.dias-uteis.pt, accessed on 1 September 2025), assuming the system and everything associated with the production (computers, printers, packaging materials, cleaning, and expendable materials) are purchased by the pharmacy. Annualized costs for fixed investments, operational consumables, and staff wages were distributed evenly across 253 working days to estimate daily expenditure. It was estimated based on medication production in an automatized MDD system for at least 700 medicated (at home and/or institutionalized) individuals considering the cheapest MDD automated machine. Considering that this automated machine includes 280 canisters and initially no auto-canisters (although it can accommodate up to 8), it is designed to serve between 150 and 800 patients. The system can store and dispense up to 280 different drugs. Additionally, it features six external trays that allow the inclusion of other drugs not not housed in the main unit, such as specific brand-name drugs, generic drugs from particular pharmaceutical labouratories, and/or orodispersible and chewable formulations that cannot remain outside their original packaging for extended periods. The preparation is made weekly for each particular or institutionalized individual.

For the purposes of this analysis, to manage a significant MDD service for patients—whether at home or institutionalized—we are proposing: pharmacist, and two pharmacy technicians (one senior and one entry-level). The pharmacist is required to review and verify the medication orders for completeness, accuracy, appropriateness, and compliance with legal standards. In addition, they conduct quality control by checking each sachet to prevent potential machine errors in drug distribution. The pharmacist also manages returns in cases where an individual passes away, leaves the institution, or stops MDD preparation. The senior pharmacy technician is responsible for machine supervision and operation, ensuring its proper functioning. This includes placing the dossge forms that cannot be loaded directly into the machine—such as large tablets, specific labouratory formulations, or supplements—in appropriate locations for preparation. The technician also refills the machine with paper and ink when necessary and corrects any errors identified by the pharmacist during the verification process (labelling adjustments). The entry-level pharmacy technician is responsible for loading the machine with the required drugs and preparing the production area for the following day. This preparation includes organizing all dosage forms that the pharmacy technician will need to place in the main unit, ensuring efficiency and order, in the next day’s production (filling). He would also clean the machine and the preparation room with alcohol and water according to the characteristics of the machine. The process of filling the machine canisters will be sourced from existing packs that require opening and hand deblistering. See Table 1 for a summary of task distribution.

## 3. Results

The cost analysis of implementing a Multidose Drug Dispensing (MDD) system in a primary care setting revealed both significant fixed and ongoing variable expenditures. Fixed implementation costs included equipment (MDD automated system), software licences, system integration, and first year maintenance. Needed equipment includes three computers, one for validation and two associated with the machine, one for selecting the institutionalized/private individuals and the other to connect directly to the machine to select the drugs that are not housed in. A printer is needed to send the medication prepared with a document that summarises all the institutionalized individuals provided for in that day. Table 2 represents the implementation costs found during 2024 and 2025. These totalled €8184.00, representing the initial investment required before operational rollout.

Monthly operational costs, which are considered variable, included labour associated with medication sorting and packaging, consumables such as unit-dose pouches, and administrative overhead. Table 3 shows monthly operational costs found during 2024 and 2025. The total monthly variable cost was €1798.25, leading to an annual variable cost of €21,579.00. When combined with the fixed costs, the total estimated cost for the first year of MDD system implementation amounted to €29,763.00.

Employees monthly costs were calculated based on legal values. A pharmacist is needed for validation, quality control, and clinical supervision; pharmacy technicians are needed for preparation, labelling, loading and operation procedures. Table 4 shows estimated values for employees’ monthly costs considering the initial salary.

The data emphasizes that although the fixed investment is substantial, the majority of expenses are attributable to ongoing operations, particularly labour. Assuming each employee receives their gross monthly salary across 14 annual payments (12 regular and 2 bonuses), the effective monthly salary over 12 months for the pharmacists is €1326.27, for the entry-level pharmacy technician is €1000.62, and for the senior pharmacy technician is €1108.33 (according to https://www.pmesalarios.pt/simulacao/custofunc accessed on 1 September of 2025).

The total cost per working day (based on 253 preparation days per year) was calculated including fixed annual costs (amortized over the first year), annual variable operational costs, and annual staff costs (adjusted for 14-month salaries paid over 12 months). It is represented in Table 5.

Considering the fixed costs (one-time implementation) as €8184.00 for the first year only, the annual variable operational costs as €21,579.00 and annual employee costs (adjusted), from adjusted monthly salaries over 12 months, as €41,222.68, total annual costs can be determined as €70,985.68. The total cost per working day (based on 253 days of MDD preparation per year) is €280.58. Labour remained the largest cost component (56.3% of daily expenditure), followed by supplies (30.8%), and fixed amortized equipment (13.0%).

The daily cost distribution of implementing and operating the MDD system shows that employee salaries represent the largest portion of daily expenses, followed by variable operational costs and fixed implementation costs.

These findings suggest that while the implementation of an MDD system requires a notable upfront investment, the sustainability of the system is heavily dependent on optimising monthly operational costs. By quantifying the fixed and operational costs associated with the service, it seeks to fill a critical evidence gap and offer practical insights for pharmacy owners, policymakers, and healthcare planners. This contribution is particularly relevant in the current context of expanding pharmaceutical care services in Europe, where robust economic data are essential to support sustainable implementation and policy integration of healthcare.

## 4. Discussion

This study presents a comprehensive cost assessment of implementing a MDD system in a community pharmacy setting. The total cost for the first year of implementation is €70,985.68. Costs were primarily determined by labour (€41,222.68/year), supplies (€21,579.00/year), and fixed equipment investments (€8184.00). These findings offer valuable insight into the financial feasibility of integrating MDD services at the pharmacy level.

While the initial investment remains notable, the major costs are associated with daily operations. Supplies—especially unit-dose pouches—represent the largest share of non-labour recurring costs, while human resources are the most significant ongoing expense, consistent with prior findings [38,39]. The updated daily operational cost of €233.91 reflects the financial commitment required for sustained service provision. However, this cost may be mitigated through workflow optimization, automation, and scaling patient volume [39,40].

Willingness to pay to pay studies, were conducted in 2014 [41] and 2023 [42]. Most individuals are willing to pay between 6 to 10 euros per month for MDD services. Considering a €6 fee per user, it would be €6 × 700 users = €5769.64 (monthly costs), which leads to €−1596.00/month. Based on a €10 monthly fee per user, a pharmacy would require a minimum of 700 users to generate a small profit margin (~€1230.36/month), and at €15 per user, the margin could increase to approximately €4730.36/month. This break-even threshold highlights the need for pharmacies to operate MDD services at scale or in cooperation with regional networks to achieve economic viability [41,42].

While initial costs may be a barrier for small or rural pharmacies, regional or national support schemes could enhance affordability and scalability. Although this study does not directly measure the clinical impact of structured dose administration (e.g., medication adherence or error reduction), these benefits remain essential topics for future exploration [43]. Furthermore, MDD integration may help reduce adverse drug events and free up clinician time, factors not quantified here but crucial for broader health economic modelling.

While this analysis was conducted in Portugal, the findings are relevant for similar pharmacy-based health systems across Europe, where MDD adoption faces comparable economic and logistical challenges. Future research should evaluate country-specific variations in labour costs, reimbursement mechanisms, and patient co-payment models to enhance global applicability. 

### Study Limitations

Several limitations must be acknowledged. The analysis was conducted from the provider’s perspective and excludes patient-related costs. Clinical outcomes, such as reductions in hospitalizations, were inferred from the literature rather than directly observed. Long-term effects, including workforce adaptation and system-wide integration challenges, were not assessed. Costs related to staff training, electricity, water, and equipment maintenance were excluded due to insufficient data. 

Moreover, the break-even analysis, which estimates viability at approximately 700 users, is based on willingness-to-pay data from 2023. This assumption may no longer reflect current market conditions. Sensitivity testing under varying payment levels, inflation rates, and patient volume scenarios would improve robustness and ensure more accurate financial forecasting.

## 5. Conclusions

The updated economic evaluation shows that MDD systems require a significant but manageable investment for community pharmacies, with daily costs estimated at €233.91. Labour remains the dominant cost driver, reinforcing the importance of workforce efficiency and service scalability. Although the initial cost may be an obstacle for some pharmacies, financial sustainability becomes attainable with more than 700 regular users and supportive reimbursement models. Given its potential to improve medication adherence and safety, MDD should be considered for broader integration into pharmacy practice. Policymakers should explore incentive mechanisms, such as direct reimbursement, bundled payments, or shared-service models, to encourage adoption, especially in regions with ageing populations and high polypharmacy prevalence. Future research should incorporate multicentre studies, real-world effectiveness assessments, and patient-reported outcomes to strengthen the evidence base. Ultimately, the economic sustainability of MDD will depend on alignment with national healthcare policies and supportive financial frameworks that reward clinical and operational performance.

## Figures and Tables

**Table 1:** Implementation costs analysed.

Cost Category	Subcategory/Item	Description/Activities	Measurement Unit/Basis	Data Source/Justification
Capital Expenditure (Fixed Costs)	Automated MDD machine	Equipment for sachet filling, sealing, and printing	Annualized over 5 years	Supplier quotations, pharmacy data
	Computer hardware and software	PC, printer, barcode reader, labeler, MDD software	Per unit	Supplier catalogues, IT budgets
Operational Costs	Packaging materials	Multidose rolls, sealing film, labels	€/sachets	Supplier catalogues
	Cleaning and consumables	Gloves, disinfectants, wipes	€/month	Pharmacy operational records
	Software licence	Annual subscription/updates	€/year	Supplier data
Labour Costs	Pharmacist	Validation, quality control, clinical supervision (can do pharmacy technician’s activity if not available)	8 h/day 40 h weekly	Portuguese Pharmaceutical Society (2024)
	Pharmacy technician(s)	Preparation, loading, labelling (can do pharmacist’s activity if not available)	8 h/day 40 h weekly	Collective agreements
Indirect Costs/Overheads	Patients served	Estimated annual MDD users	—	Real-world pharmacy data
	Working days	MDD preparation days per year	—	National pharmacy calendar (online calculator of effective working days for 2025)
	Sachets produced	Estimated daily sachet output	—	Machine specifications

**Table 2:** Fixed costs (amortized over 253 days).

Category	Annual Cost (€)	Daily Cost (€)
MDD automated system (software licences for 1 year, system integration and first year maintenance)	7950.00	31.42
Computers (3 × €350) (5 years)	210.00 1050.00—5 year guarantee	0.83
Printer (5 years)	24.00 120.00—5 year guarantee	0.09
Total fixed costs	8 184.00	32.34

All the values were consulted from internet websites based on publicly available data from several pharmacy distributors operating in Portugal, as well as general online retail platforms accessible worldwide.

**Table 3:** Monthly operational costs (amortized over 253 days).

Description	Minimum Value (€)	Quantity	Daily Cost (€)	Monthly Cost (€)
Unit-dose pouches Ink Latex gloves	43.50	29	59.83	1261.50
	15.30	29	21.05	443.70
	0.070	500	1.66	35.00
White disposable caps Surgical mask type IIR	0.058	100	0.28	5.80
	0.062	500	1.47	31.00
Shoe covers	0.085	250	1.01	21.25
Total operational costs (€)			81.74	1798.25

All the values were consulted in January 2024 and February 2025. It was considered a month with 22 days of preparation. Annual cost calculated based on a year of 365 days with 253 days of preparation in a year. It was considered 12 months/year.

**Table 4:** Employees’ monthly costs (amortized over 253 days).

Employee Type	Annual Cost (€)	Daily Cost (€)
Pharmacist	15,915.24	62.91
Senior pharmacy technician	13,300.00	52.57
Entry-level pharmacy technician	12,007.44	47.46
Total salaries	41,222.68	162.94

All the values were consulted on internet websites (https://bte.gep.msess.gov.pt/completos/2025/bte16_2025.pdf) accessed on 1 June 2025. It was considered a month with 22 days of preparation. Annual cost calculated based on a year of 365 days with 253 days of preparation in a year. It was considered 12 months/year.

**Table 5:** Total Cost Per Working Day (253 Days/Year).

Cost Category	Daily Cost (€)
Fixed implementation	32.34
Variable operational	85.30
Employee salaries	162.94
TOTAL	280.58

Electricity and space costs were not considered because these values are not available.

## Data Availability

No new data were created or analyzed in this study. Data sharing is not applicable to this article.
